# Visualization of epithelial-mesenchymal transition in an inflammatory microenvironment–colorectal cancer network

**DOI:** 10.1038/s41598-019-52816-z

**Published:** 2019-11-08

**Authors:** Takeshi Ieda, Hiroshi Tazawa, Hiroki Okabayashi, Shuya Yano, Kunitoshi Shigeyasu, Shinji Kuroda, Toshiaki Ohara, Kazuhiro Noma, Hiroyuki Kishimoto, Masahiko Nishizaki, Shunsuke Kagawa, Yasuhiro Shirakawa, Takashi Saitou, Takeshi Imamura, Toshiyoshi Fujiwara

**Affiliations:** 1 Department of Gastroenterological Surgery, Okayama University Graduate School of Medicine, Dentistry and Pharmaceutical Sciences, Okayama, 700-8558 Japan; 2 Pathology & Experimental Medicine, Okayama University Graduate School of Medicine, Dentistry and Pharmaceutical Sciences, Okayama, 700-8558 Japan; 3 Center for Innovative Clinical Medicine, Okayama University Hospital, Okayama, 700-8558 Japan; 4 Minimally Invasive Therapy Center, Okayama University Hospital, Okayama, 700-8558 Japan; 5 Department of Molecular Medicine for Pathogenesis, Ehime University Graduate School of Medicine, Ehime, 791-0295 Japan

**Keywords:** Colon cancer, Cancer imaging

## Abstract

Epithelial-mesenchymal transition (EMT) is a biological process by which epithelial cells acquire mesenchymal characteristics. In malignant tumors, EMT is crucial for acquisition of a mesenchymal phenotype with invasive and metastatic properties, leading to tumor progression. An inflammatory microenvironment is thought to be responsible for the development and progression of colorectal cancer (CRC); however, the precise role of inflammatory microenvironments in EMT-related CRC progression remains unclear. Here, we show the spatiotemporal visualization of CRC cells undergoing EMT using a fluorescence-guided EMT imaging system in which the mesenchymal vimentin promoter drives red fluorescent protein (RFP) expression. An inflammatory microenvironment including TNF-α, IL-1β, and cytokine-secreting inflammatory macrophages induced RFP expression in association with the EMT phenotype in CRC cells. *In vivo* experiments further demonstrated the distribution of RFP-positive CRC cells in rectal and metastatic tumors. Our data suggest that the EMT imaging system described here is a powerful tool for monitoring EMT in inflammatory microenvironment–CRC networks.

## Introduction

Epithelial-mesenchymal transition (EMT) is a biological process in which epithelial cells acquire the mesenchymal phenotype in embryonic development, tissue fibrosis, and tumor progression^1–3^. EMT is induced in various types of normal cells during gastrulation or as a physiologic response to tissue injury^4^. During wound healing, tissue fibrosis is induced through an EMT process that enables the conversion of epithelial cells into mesenchymal cells. In tumor tissues, EMT induces the mesenchymal phenotype with malignant properties associated with migration, invasion, and metastasis^5^. EMT signatures are highly associated with poor prognosis in patients with various types of cancers, such as lung^6^, breast^7^, esophagus^8^, stomach^9^, colon^10^, and pancreas^11^. However, recent reports have suggested that EMT is associated with chemoresistance (but not metastatic) processes in animal models of lung and pancreatic cancer^12,13^. Thus, the precise role of EMT during tumor progression is incompletely understood.

The tumor microenvironment plays a critical role in tumor progression^14^. An inflammatory microenvironment is thought to be responsible for the development and progression of colorectal cancer (CRC)^15^. An inflammatory microenvironment consists of immune cells, cytokines, growth factors, stromal cells, and extracellular matrix (ECM)^15^. In the acute inflammatory phase^16–19^, infiltration of immune cells, such as macrophages and neutrophils, contributes to the accumulation of pro-inflammatory cytokines, including tumor necrosis factor–α (TNF-α), interleukin-1β (IL-1β), and transforming growth factor–β (TGF-β). By contrast, in the chronic inflammatory phase^16–19^, several growth factors, including epidermal growth factor (EGF), basic fibroblast growth factor (bFGF), and hepatocyte growth factor (HGF), also accumulate with proliferation of stromal fibroblasts in the ECM. However, it remains unclear how an inflammatory microenvironment induces EMT during CRC progression. To resolve this issue, the development of an EMT imaging system would be a promising approach for exploring the underlying mechanism of EMT in the interplay between an inflammatory microenvironment and CRC.

Fluorescent proteins are widely used to investigate the behavior of tumor cells^20,21^. Fluorescence-guided imaging technologies enable the visualization of tumor cells in primary and metastatic regions. Recent studies using EMT-dependent fluorescent probes demonstrated the visualization of tumor cells undergoing EMT. Fischer *et al*. developed a mesenchymal marker–specific Cre-mediated fluorescent switching system^12,22^ in which the fibroblast-specific protein 1 promoter drives the Cre-lox recombination system to induce expression of green fluorescent protein (GFP) in cells undergoing EMT. However, this switching is not reversible, and GFP-positive tumor cells can include epithelial-type cells that undergo EMT. By contrast, Choi *et al*. developed a molecular beacon based on a non-coding microRNA (miRNA) 200a binding sequence^23^. This binding is reversible, in contrast to the mesenchymal marker–specific imaging system. Thus, a reversible, mesenchymal marker–specific fluorescent probe is needed to explore the induction of EMT in tumor cells.

EMT is orchestrated by several intrinsic factors, such as EMT-related transcription factors (EMT-TFs) and EMT-related miRNAs (EMT-miRNAs)^5,24,25^. At the transcriptional level, EMT is induced by the activation of EMT-TFs, including proteins of the SNAIL and zinc finger E-box–binding homeobox (ZEB) families^5,26^, which bind to the promoter region of downstream target genes. EMT-TFs downregulate the expression of the gene encoding the epithelial marker E-cadherin (CDH1), whereas they upregulate the expression of the gene encoding the mesenchymal marker vimentin (VIM). By contrast, EMT is inhibited at the post-transcriptional level by the activation of EMT-miRNAs, including those of the miR-34 and miR-200 families^5,27,28^, which bind to the 3′-untranslated region (3′-UTR) of downstream target genes. EMT-miRNAs downregulate the expression of EMT-TFs and mesenchymal marker genes. Therefore, EMT involves a complex regulatory network consisting of EMT-TFs, EMT-miRNAs, and epithelial and mesenchymal marker genes.

In the present study, we developed a fluorescence-guided EMT imaging system in which the promoter region and 3′-UTR of the mesenchymal *VIM* gene were used to reversibly induce EMT-dependent expression of red fluorescent protein (RFP) in human CRC cells. The role of an inflammatory microenvironment created by mediators such as inflammatory cytokines, growth factors, and macrophages was evaluated by examining the induction of EMT-dependent RFP expression in human CRC cells. Furthermore, the distribution of CRC cells undergoing EMT in tumor tissues was analyzed *in vivo* using primary and metastatic xenograft tumors.

## Results

### Development of VIM promoter–driven RFP expression system using CRC cells

**Figure 1:**
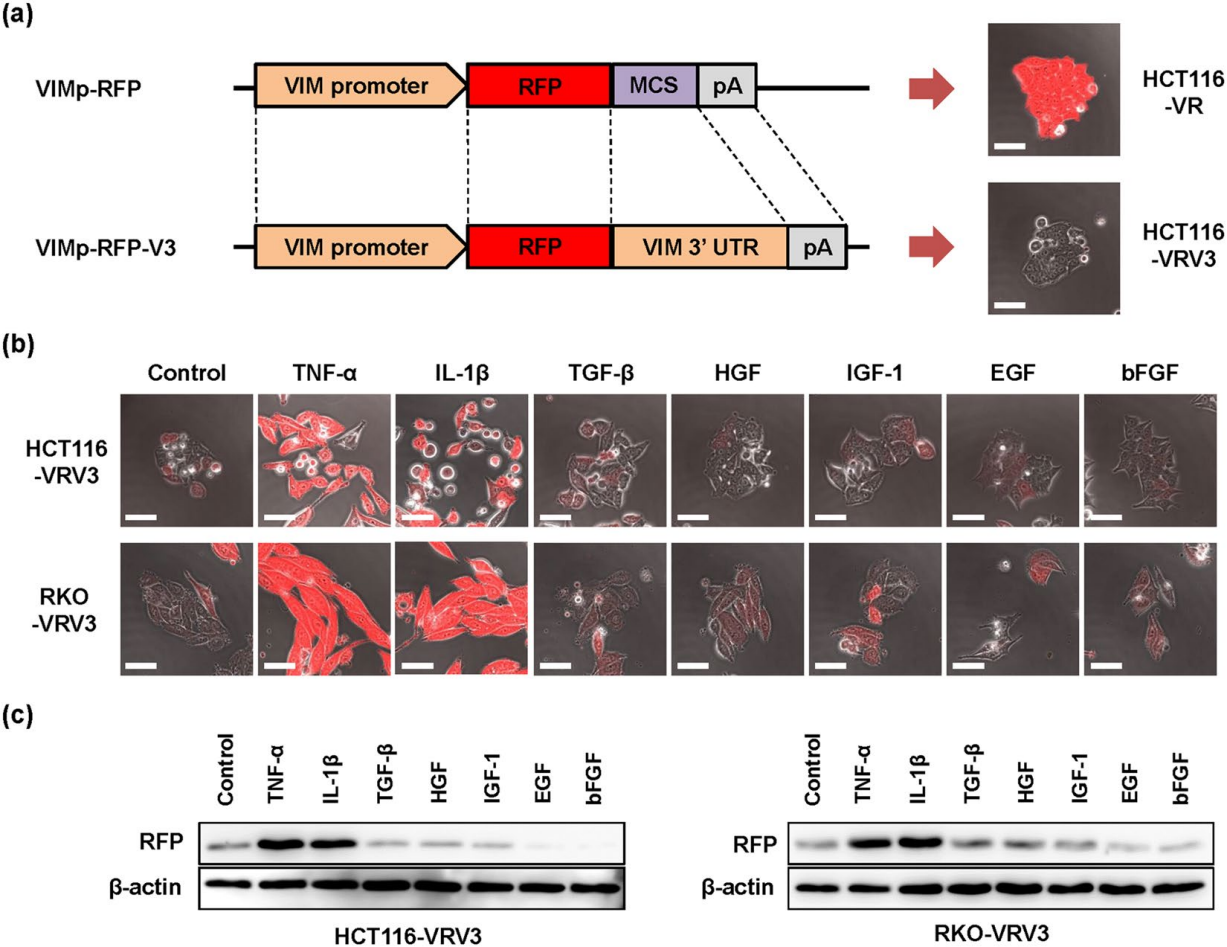
Development of *VIM* promoter–driven RFP expression system using CRC cell lines. (**a**) Structure for *VIM* promoter–driven RFP expression vector without or with *VIM* 3′-UTR, VR or VRV3 vector, respectively. Photographs of HCT116 cells stably transfected with VR and VRV3 vectors, HCT116-VR and HCT116-VRV3. Scale bars: 50 μm. MCS, multi-cloning site. (**b**) Photographs of HCT116-VRV3 and RKO-VRV3 cells treated without or with TNF-α (20 ng/ml), IL-1β (1 ng/ml), TGF-β (10 ng/ml), HGF (50 ng/ml), IGF-1 (20 ng/ml), EGF (20 ng/ml), or bFGF (10 ng/ml) for 48 h. Scale bars: 50 μm. (**c**) expression of RFP in HCT116-VRV3 and RKO-VRV3 cells treated without or with TNF-α (20 ng/ml), IL-1β (1 ng/ml), TGF-β (10 ng/ml), HGF (50 ng/ml), IGF-1 (20 ng/ml), EGF (20 ng/ml), or bFGF (10 ng/ml) for 48 h. β-actin was used as a loading control.

To visualize the dynamic changes associated with EMT in human CRC cells, we sought to develop an EMT-dependent fluorescent cell imaging system. As EMT is positively regulated by EMT-TFs that bind to the promoter region of the target gene and negatively regulated by EMT-miRNAs that bind to the 3′-UTR of the target gene, the promoter region and 3′-UTR of the gene encoding the mesenchymal marker VIM were cloned from human normal fibroblast WI-38 cells. Using the promoter region and 3′-UTR of the *VIM* gene, we constructed two different *VIM* promoter–driven RFP expression vectors, with and without the *VIM* 3′-UTR, designated VRV3 and VR, respectively (Fig. 1a and Supplementary Information Fig. 1).

Previous reports have shown that HCT116 human CRC cells exhibit an epithelial phenotype and that EMT is induced in these cells by treatment with inflammatory cytokines such as TNF-α^29^ or IL-1β^30^. Therefore, we next established two types of HCT116 transfectants, HCT116-VRV3 and HCT116-VR, which were stably transfected with the VRV3 vector carrying the *VIM* 3′-UTR and the VR vector lacking the *VIM* 3′-UTR, respectively, from 5 candidate clones (Supplementary Information Fig. 2). In the absence of EMT inducer, *VIM* 3′-UTR–lacking HCT116-VR cells exhibited RFP expression (Fig. 1a and Supplementary Information Fig. 2), indicating EMT-independent RFP expression. By contrast, *VIM* 3′-UTR–carrying HCT116-VRV3 cells exhibited RFP expression when treated with TNF-α or IL-1β for 48 h (Fig. 1b and Supplementary Information Fig. 2), suggesting EMT-dependent RFP expression.

Next, to demonstrate RFP expression in another cell type, RKO-VRV3 human CRC cells stably transfected with the VRV3 vector carrying the 3′-UTR were treated with TNF-α or IL-1β for 48 h (Fig. 1b). However, other types of cytokines and growth factors, including TGF-β, EGF, bFGF, HGF, and IGF-1, did not induce RFP expression in HCT116-VRV3 or RKO-VRV3 cells (Fig. 1b). Western blot analyses also demonstrated that, consistent with fluorescence imaging analyses, RFP expression was upregulated in HCT116-VRV3 and RKO-VRV3 cells only when treated with TNF-α or IL-1β (Fig. 1c). In the absence of EMT inducers, HCT116-VRV3 and RKO-VRV3 cells exhibited characteristics similar to the parental cells in terms of morphology and proliferation (Supplementary Information Fig. 3). These results indicate that HCT116-VRV3 and RKO-VRV3 cells would be useful tools for evaluating the dynamic changes associated with EMT in inflammatory microenvironments induced by factors such as TNF-α and IL-1β.

### Induction of RFP expression in association with EMT phenotype in CRC cells

**Figure 2:**
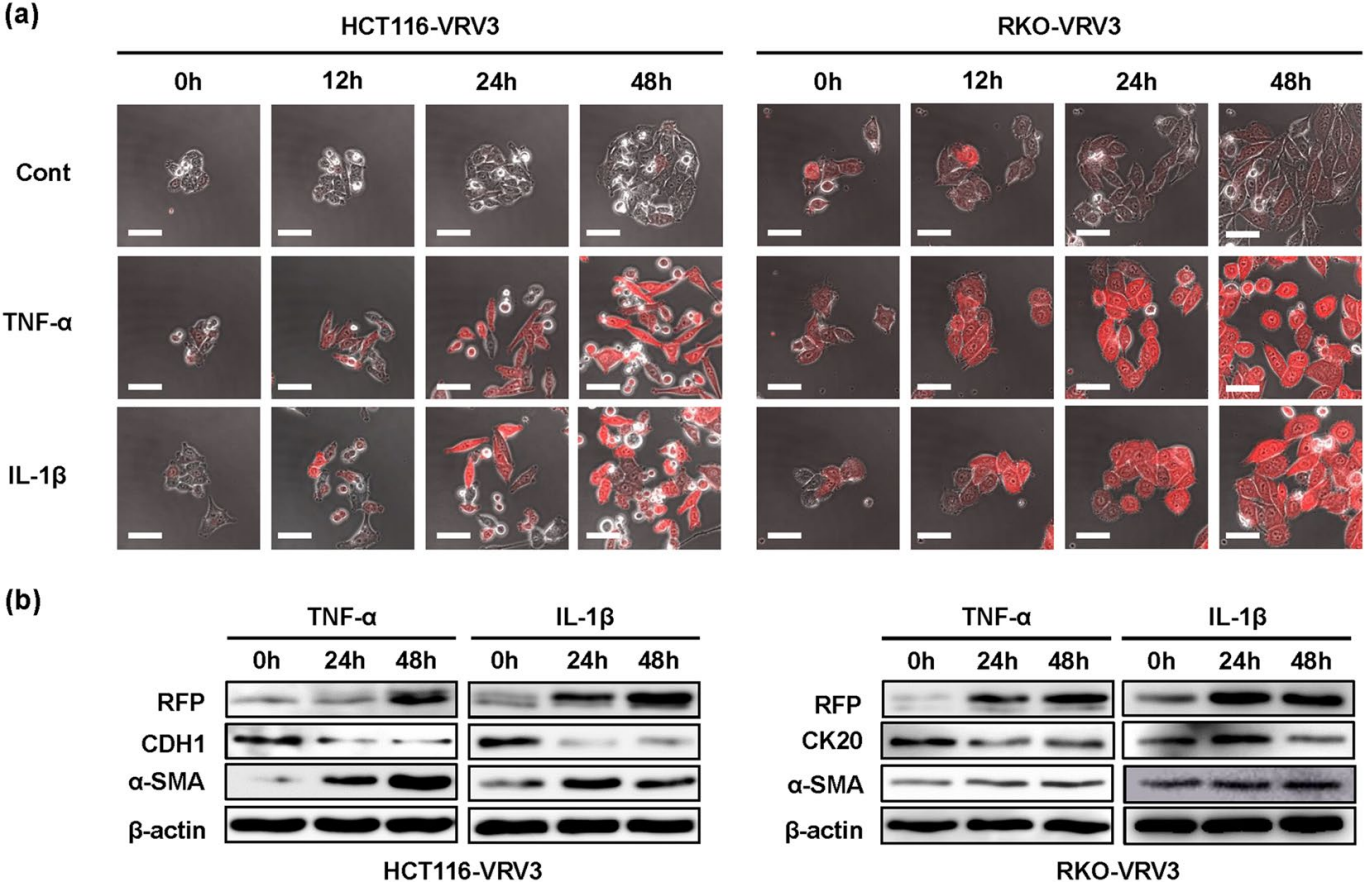
TNF-α– and IL-1β–mediated induction of RFP expression and EMT phenotype. (**a**) Photographs of HCT116-VRV3 and RKO-VRV3 cells treated with TNF-α (20 ng/ml) or IL-1β (1 ng/ml) for 48 h. Scale bars: 50 μm. (**b**) Expression of RFP, epithelial markers (CDH1 and CK20), and a mesenchymal marker (α-SMA) in HCT116-VRV3 and RKO-VRV3 cells treated with TNF-α (20 ng/ml) or IL-1β (1 ng/ml) for 48 h. β-actin was used as a loading control.

To confirm the relationship between RFP expression and the EMT phenotype in HCT116-VRV3 and RKO-VRV3 cells, we examined morphologic changes using time-lapse imaging and levels of RFP and epithelial and mesenchymal marker expression using western blot and real-time RT-PCR analysis. HCT116-VRV3 and RKO-VRV3 cells treated with TNF-α or IL-1β exhibited time-dependent changes in morphology, characterized by spindle shape and low number of cell-cell attachments in association with RFP expression, compared to non-treated control cells (Fig. 2a and Supplementary Movies 1–6). Increased RFP expression was further confirmed by flow cytometry and microplate reader (Supplementary Information Figs 4 and 5). Consistent with induction of RFP expression, expression of the mesenchymal markers α-SMA and VIM in HCT116-VRV3 and RKO-VRV3 cells increased, whereas expression of the epithelial markers CDH1 and CK20 decreased (Fig. 2b and Supplementary Information Figs 4 and 5). Moreover, the migration and invasion capabilities of HCT116-VRV3 and RKO-VRV3 cells treated with TNF-α or IL-1β increased significantly (Supplementary Information Figs 4 and 5). These results suggest that inflammatory cytokine–induced RFP expression is associated with the EMT phenotype in CRC cells.

### Plasticity of EMT-dependent RFP expression in CRC cells

**Figure 3:**
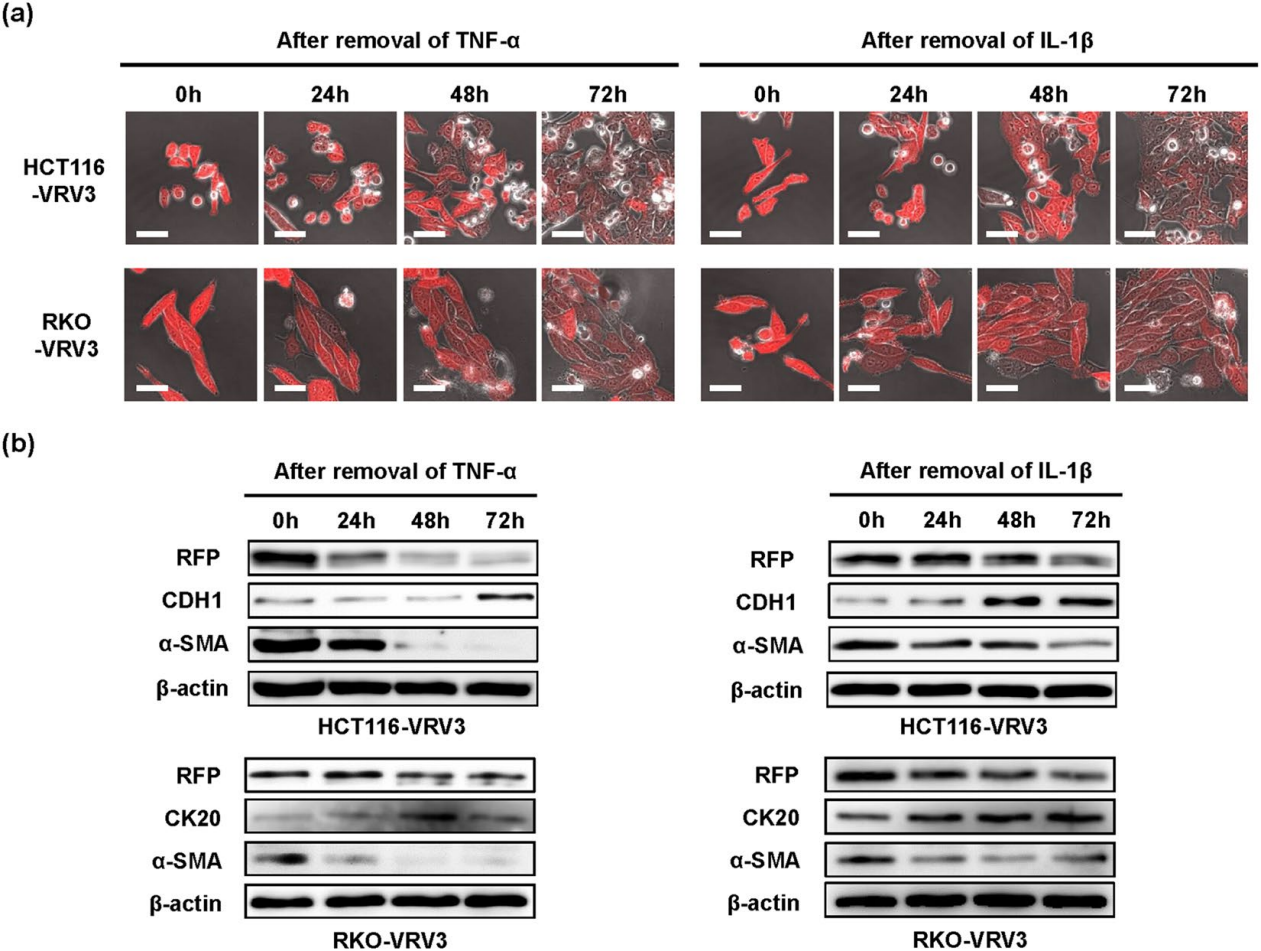
Reversibility of TNF-α– and IL-1β–induced RFP expression and EMT phenotype. (**a**) Photographs of HCT116-VRV3 and RKO-VRV3 cells after removal of cytokines following treatment with TNF-α (20 ng/ml) or IL-1β (1 ng/ml) for 48 h. Scale bars: 50 μm. (**b**) Expression of RFP, epithelial markers (CDH1 and CK20), and a mesenchymal marker (α-SMA) in HCT116-VRV3 and RKO-VRV3 cells after removal of cytokines following treatment with TNF-α (20 ng/ml) or IL-1β (1 ng/ml) for 48 h. β-actin was used as a loading control.

To investigate the reversibility of EMT-dependent RFP expression, HCT116-VRV3 and RKO-VRV3 cells were treated with TNF-α or IL-1β for 48 h. After removal of the inflammatory cytokine, RFP expression and morphologic changes were analyzed over a 72-h period using time-lapse imaging. Inflammatory cytokine–induced RFP expression and spindle-shaped morphologic changes gradually diminished in HCT116-VRV3 and RKO-VRV3 cells after the removal of inflammatory cytokine, and the number of cell-cell attachments increased (Fig. 3a). Western blot analyses demonstrated decreased expression of RFP and the mesenchymal marker α-SMA in HCT116-VRV3 and RKO-VRV3 cells after removal of TNF-α or IL-1β, whereas expression of the epithelial markers CDH1 and CK20 increased (Fig. 3b). Moreover, anti–TNF-α neutralizing antibody and anti–IL-β neutralizing antibody treatment was associated with a dose-dependent reversal of TNF-α– and IL-β–induced RFP expression, respectively, in HCT116-VRV3 and RKO-VRV3 cells (Supplementary Information Figs 6 and 7). These results suggest that EMT-dependent RFP expression in CRC cells is reversibly regulated by the inflammatory cytokines TNF-α and IL-β.

### Induction of RFP expression in CRC cells co-cultured with inflammatory macrophages

**Figure 4:**
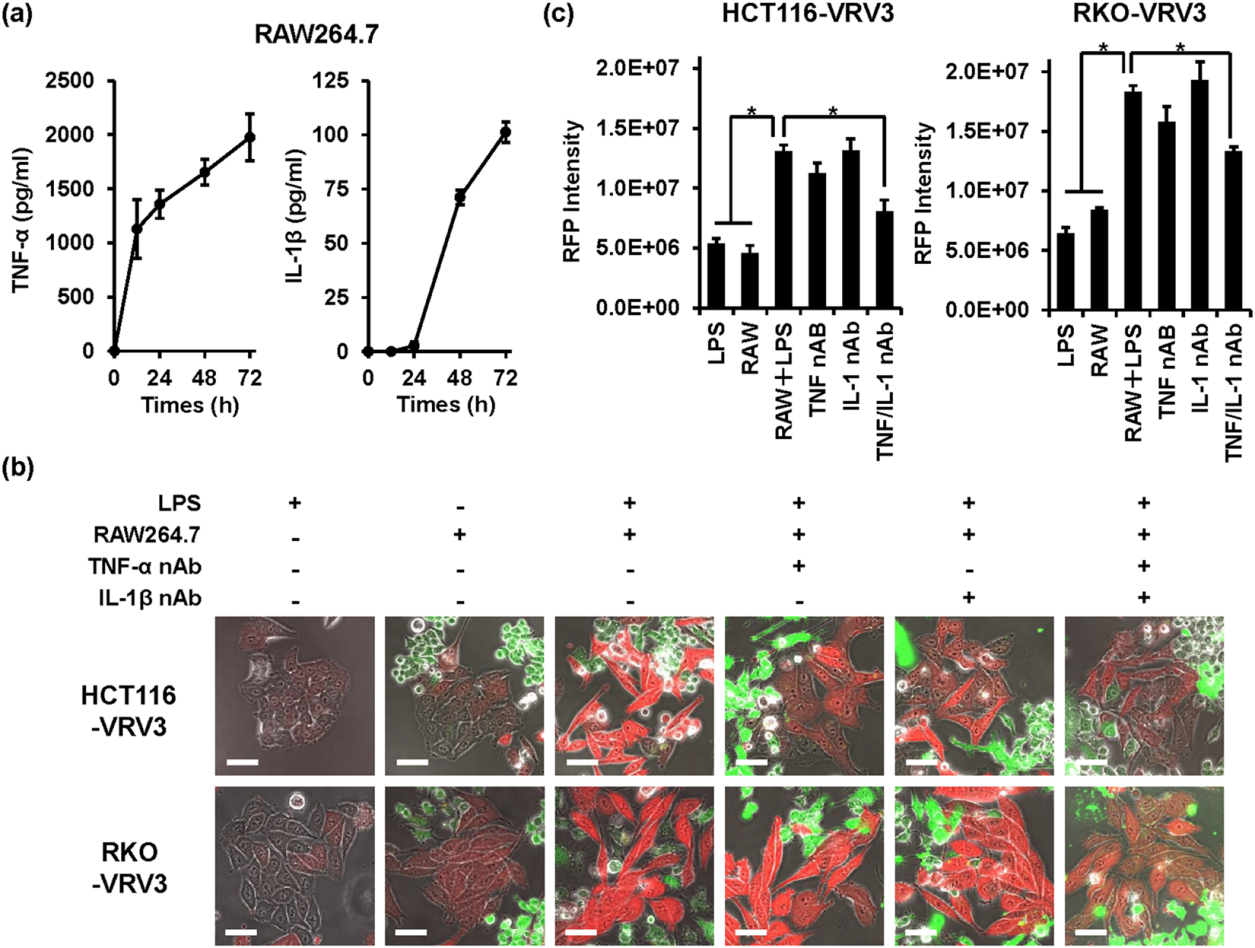
Direct co-culture of CRC cells and RAW264.7 cells. (**a**) Quantification of TNF-α and IL-1β secreted by RAW264.7 cells after stimulation with LPS (200 ng/ml) for 72 h. Data are expressed as mean ± SD (n = 3). (**b**) Photographs of HCT116-VRV3 and RKO-VRV3 cells in direct co-culture with RAW264.7 cells. LPS (200 ng/ml) was administered to induce the secretion of TNF-α and IL-1β by RAW264.7 cells. Anti–TNF-α neutralizing antibody (100 ng/ml) and anti–IL-1β neutralizing antibody (500 ng/ml) were administered to inhibit the effects of TNF-α and IL-1β secreted by RAW264.7 cells. RAW264.7 cells were stained with CellTracker Green to distinguish them from HCT116-VRV3 and RKO-VRV3 cells. Scale bars: 50 μm. (**c**) Quantification of RFP expression in HCT116-VRV3 and RKO-VRV3 cells directly co-cultured with RAW264.7 cells. *P < 0.05.

Previous reports have shown that RAW264.7 mouse macrophages stimulated with LPS exhibit the pro-inflammatory M1 phenotype associated with secretion of inflammatory cytokines such as TNF-α^31^ and IL-1β^32^. To mimic the interplay between CRC cells and an inflammatory microenvironment, we co-cultured CRC cells with LPS-stimulated RAW264.7 cells. Administration of LPS induced the secretion of TNF-α and IL-1β by RAW264.7 cells in a time-dependent manner (Fig. 4a). In direct co-culture of HCT116-VRV3 and RKO-VRV3 CRC cells and RAW264.7 cells, LPS-stimulated RAW264.7 cells significantly induced expression of RFP in the cancer cells, although non-stimulated RAW264.7 cells or LPS alone did not (Fig. 4b,c). Administration of anti–TNF-α neutralizing antibody or anti–IL-β neutralizing antibody alone did not suppress RFP expression (Fig. 4b,c). However, administration of both antibodies significantly suppressed RFP expression in both HCT116-VRV3 and RKO-VRV3 cells (Fig. 4b,c), suggesting that TNF-α and IL-β play a significant role in EMT-dependent induction of RFP expression. Moreover, even in indirect co-culture of HCT116-VRV3 and RKO-VRV3 CRC cells and RAW264.7 macrophages, LPS-stimulated RAW264.7 cells efficiently induced RFP expression in the cancer cells in a time-dependent manner (Supplementary Information Fig. 8). By contrast, IL-4–stimulated RAW264.7 cells, which exhibit the pro-tumoral M2 phenotype, did not induce RFP expression in HCT116-VRV3 cells (Supplementary Information Fig. 9). These results suggest that inflammatory macrophages have the potential to induce EMT-dependent RFP expression in CRC cells via secretion of TNF-α and IL-1β.

### Distribution of RFP-positive CRC cells and inflammatory macrophages in primary and metastatic tumors

**Figure 5:**
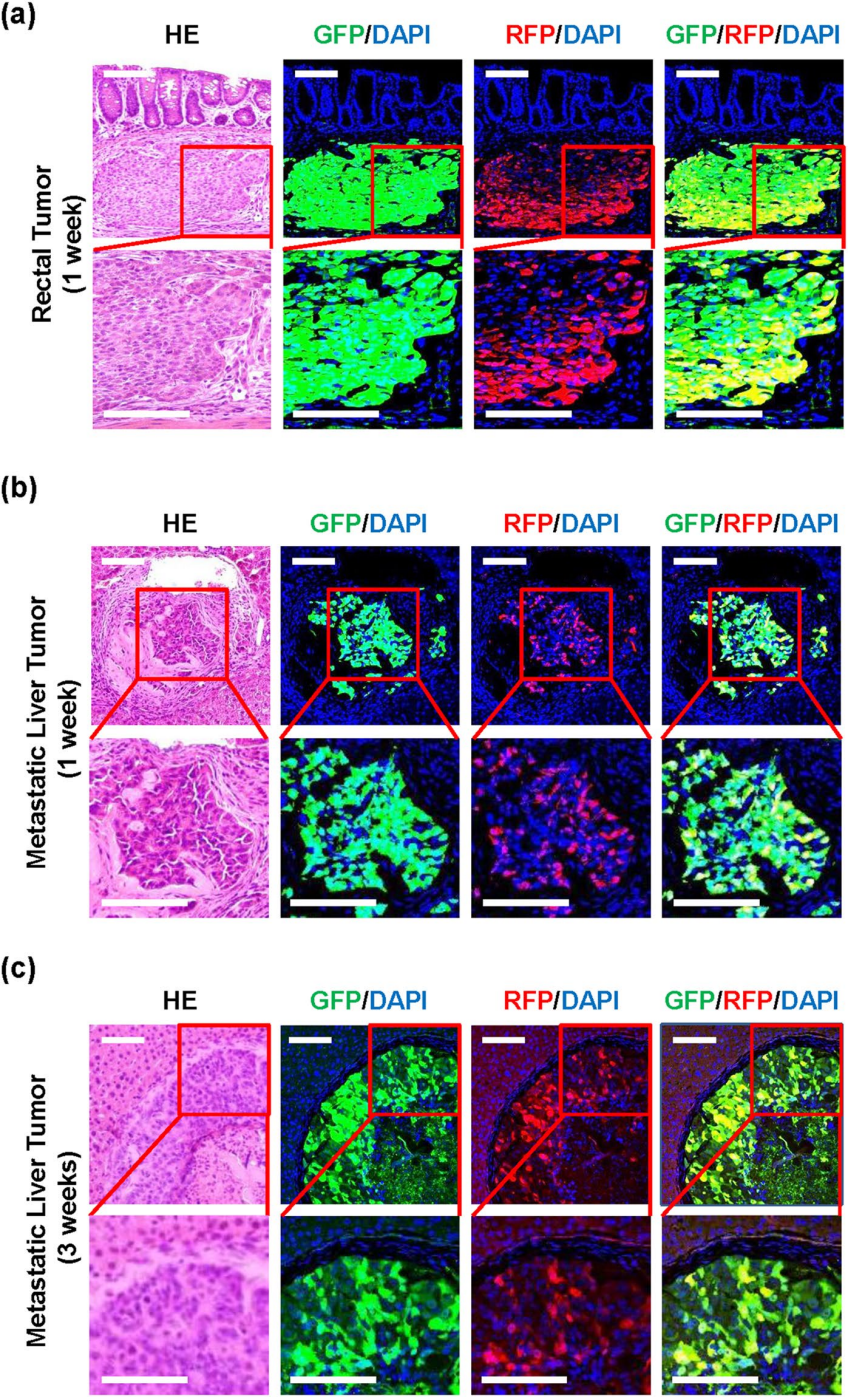
Localization of RFP-positive HCT116-VRV3 cells in tumor tissues. (**a**–**c**) Immunohistochemistry for GFP and RFP in rectal tumor (**a**), metastatic liver tumor at 1 week (**b**) and metastatic liver tumor at 3 weeks (**c**). Nuclei were stained with DAPI. HE, hematoxylin-eosin. Scale bars: 100 μm.

To investigate the *in vivo* distribution of RFP-positive HCT116 cells undergoing EMT, we used CRC xenograft animal models of rectal and metastatic liver tumors^33,34^. HCT116-VRV3-GFP cells stably transfected with GFP expression vector were used to visualize GFP-positive HCT116-VRV3 cells in inoculated tissues. HCT116-VRV3-GFP cells were inoculated into submucosal tissues of rectum for rectal tumors and subcapsular tissues of spleen for metastatic liver tumors, although inoculated tissues were not physiological. Immunohistochemical analysis demonstrated localization of RFP-positive CRC cells undergoing EMT in invasive areas of GFP-positive rectal tumors at 1 week after inoculation (Fig. 5a). When we analyzed metastatic liver tumors at 1 and 3 weeks after inoculation, RFP-positive CRC cells were also observed in metastatic liver tumors (Fig. 5b,c). These results suggest that EMT is induced in rectal and metastatic liver tumors.

**Figure 6:**
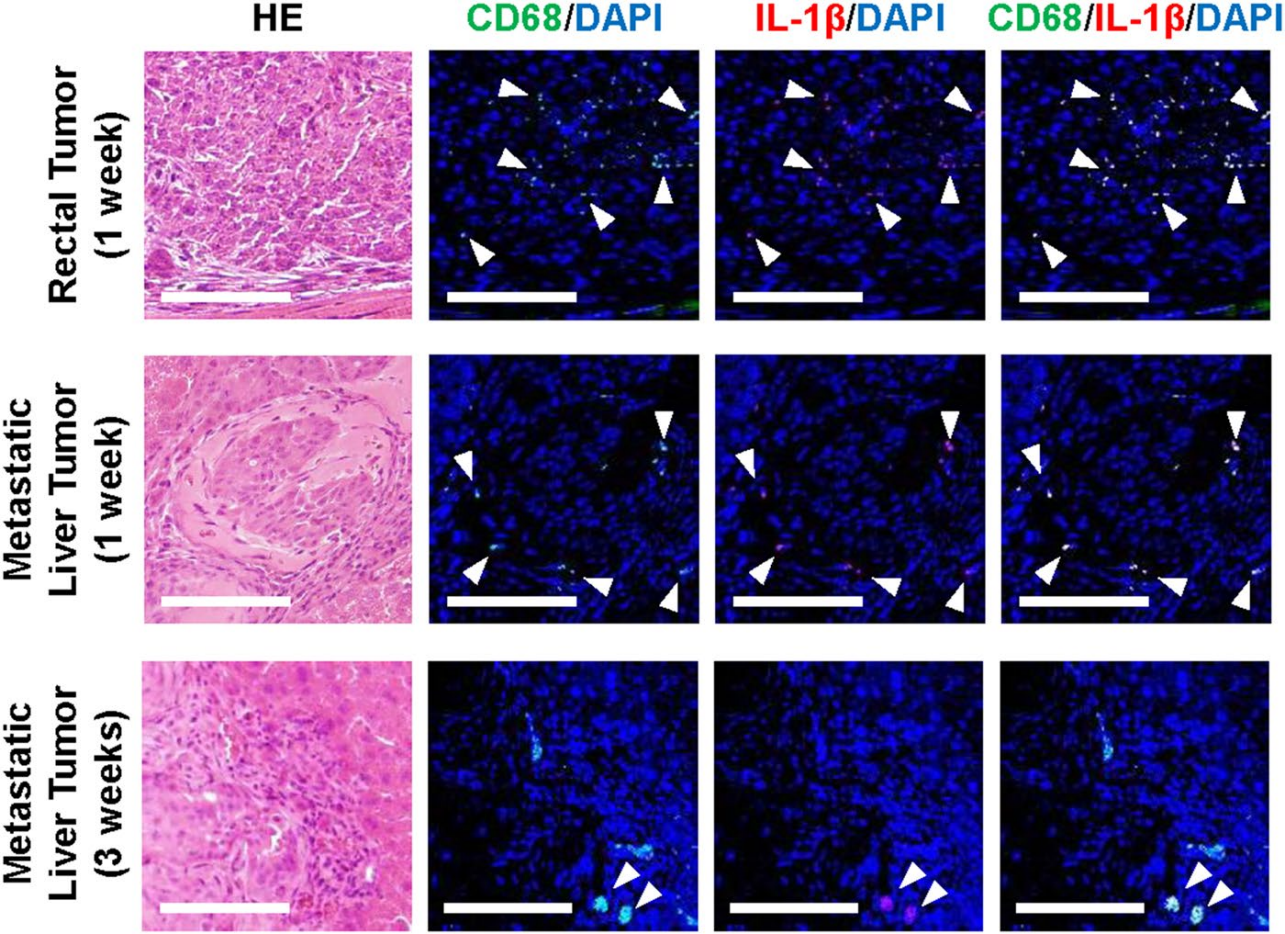
Localization of CD68-positive inflammatory macrophages in tumor tissues. Immunohistochemistry for CD68 and IL-1β in rectal tumor (1 week) and metastatic liver tumors (1 and 3 weeks). White head arrows indicate CD68-positive and IL-1β–positive cells. Scale bars: 100 μm.

We next investigated whether an inflammatory microenvironment is associated with EMT of CRC cells. Immunohistochemical analysis demonstrated localization of CD68-positive macrophages expressing IL-1β (but not TNF-α) in rectal and metastatic liver tumors, consistent with the distribution of CRC cells undergoing EMT (Fig. 6). These results suggest that cytokine-secreting inflammatory macrophages are involved in inducing EMT of CRC cells.

## Discussion

The induction of EMT converts epithelial cancer cells to a mesenchymal phenotype characterized by migration, invasion, and chemoresistance. However, the underlying mechanism of EMT induction during tumor progression is incompletely understood. In this study, we developed a novel fluorescence-guided EMT imaging approach for CRC using a mesenchymal *VIM* promoter–driven RFP expression vector containing the *VIM* 3′-UTR. An inflammatory microenvironment characterized by the presence of factors such as TNF-α, IL-1β, and cytokine-secreting macrophages induced reversible RFP expression in association with an EMT-related malignant phenotype in HCT116 and RKO cells. *In vivo* experiments demonstrated the presence of RFP-positive CRC cells undergoing EMT in rectal and metastatic liver tumors adjacent to IL-1β–expressing inflammatory macrophages. Thus, although *in vivo* experiments using several clones of HCT116-VRV3 cells are needed because one clone was used in this study, the EMT imaging system we developed is a promising approach for exploring the underlying mechanism of EMT induction in inflammatory microenvironment–related CRC progression.

The promoter region and 3′-UTR of the *VIM* gene were found to be critical for inducing EMT-dependent RFP expression in HCT116 and RKO cells, indicating the involvement of EMT-TFs and EMT-miRNAs in the regulation of EMT. Previous reports demonstrated that TNF-α– and IL-1β–induced ΕΜΤ in HCT116 cells is associated with activation of the EMT-TFs Snail and Zeb1, respectively^29,30^. By contrast, the EMT-miRNAs of the miR-200 family reportedly determine the epithelial and mesenchymal phenotypes in NCI60 cancer cells; epithelial type HCT116 cells show high expression of members of the miR-200 family^35^. Reduced expression of miR-200 family molecules can induce EMT in HCT116 cells through activation of ZEB family proteins^35^. Moreover, downregulation of miR-200a expression is associated with EMT signatures in CRC tissues^36^ and inflamed tissues of inflammatory bowel disease (IBD)^37^. Thus, further experiments exploring the complex interaction between the EMT network and inflammatory cytokines, EMT-TFs, and EMT-miRNAs are warranted.

Tumor-associated macrophages (TAMs) have emerged as a critical factor in tumor progression^38,39^. Although anti-inflammatory M2 macrophages function as TAMs, whether pro-inflammatory M1 macrophages play a role in tumor progression remains unclear. In this study, M1 macrophages (but not M2 macrophages) exhibited a potential to induce EMT in HCT116 and RKO cells through secretion of TNF-α and IL-1β, consistent with previous reports of EMT induction in HCT116 cells treated with TNF-α^29^ and IL-1β^30^. *In vivo* experiments further demonstrated that IL-β–expressing inflammatory macrophages are involved in inducing EMT in HCT116 cells in rectal and metastatic liver tumors. Inflammatory macrophages have been shown to induce EMT in fibrosis in response to tissue injury. Indeed, EMT signatures can be observed in fibrotic tissues associated with IBD^40^. The secretion of IL-1β by inflammatory macrophages is tightly regulated by the activation of inflammasome signaling, which is a central factor in the pathogenesis of IBD^41^. However, recent reports have shown that M2 macrophages also exhibit the secretion of TNF-α and IL-1β, leading to EMT induction in pancreatic ductal adenocarcinoma cells^42^, esophageal squamous cell carcinoma cells^43^ and hepatocellular carcinoma cells^44^. Therefore, further experiments are warranted to characterize IL-1β-expressing macrophages in tumor tissues.

It is worth noting that in addition to TNF-α and IL-1β, TGF-β can also induce EMT^45^. Administration of TGF-β did not induce EMT-dependent RFP expression in HCT116 and RKO cells. It has been shown that CRC cells with microsatellite instability (MSI) that have a mutant TGF-β receptor type II are less sensitive to TGF-β–induced EMT than CRC cells with microsatellite stability that have intact TGF-β receptor type II^46^. Indeed, HCT116 and RKO cells exhibit MSI and do not express TGF-β receptor type II^47^. Liu *et al*. demonstrated that restoration of TGF-β receptor type II expression in HCT116 cells increased sensitivity to TGF-β in terms of inducing the EMT phenotype and metastatic potential^48^. As TNF-α synergistically enhanced TGF-β–induced EMT in a CRC organoid culture model^49^, the use of CRC cells responsive to TGF-β, TNF-α, and IL-1β may be more suitable for exploring the underlying mechanism of inflammatory microenvironment–mediated EMT induction in CRC.

Recent evidence suggests that CRC can be classified into four consensus molecular subtypes (CMSs), based on comprehensive analyses of DNA, RNA, and protein gene expression profiles^50^. CMS4 is highly associated with EMT signatures and worse prognosis compared to the other CMSs^50^. CMS4 CRC with EMT signatures is thought to be associated with stromal activation, immunosuppression, inflammation, and angiogenesis^51^. Significant infiltration of CD68-positive macrophages is also characteristic of CMS4 CRC^52^. A recent report examining 34 human CRC cell lines indicated that HCT116 and RKO cells are CMS4^53^. As the precise mechanism of EMT regulation in CMS4 CRC remains unclear, our EMT imaging system using HCT116 and RKO CMS4 CRC cells may be a useful platform for evaluating the underlying mechanism of EMT regulation in the CMS4 CRC–inflammatory microenvironment network.

In conclusion, we developed an EMT-dependent fluorescent cell imaging system using human CRC cells. An inflammatory microenvironment characterized by the presence of TNF-α, IL-1β, and cytokine-secreting macrophages was found to be crucial for inducing EMT in CRC cells. Although recent reports suggest that the role of EMT in the metastatic process has yet to be fully elucidated, EMT is induced by inflammatory cytokines and infiltrating macrophages in primary and metastatic tumors. This fluorescence-guided EMT imaging system we developed may offer new perspectives in understanding the underlying mechanism of EMT regulation in the interplay between CRC and inflammatory microenvironments.

## Materials and Methods

### Cell lines

Human HCT116 and RKO CRC cells and RAW264.7 mouse macrophages were obtained from the American Type Culture Collection (Manassas, VA, USA). HCT116 cells were maintained in MaCoy’s 5 A medium. RKO and RAW264.7 cells were cultured in Eagle’s Minimum Essential Medium and Dulbecco’s modified Eagle’s medium, respectively. All media were supplemented with 10% fetal bovine serum (FBS), 100 U/ml penicillin, and 100 mg/ml streptomycin. The cells were routinely maintained at 37 °C in a humidified atmosphere with 5% CO_2_. HCT116 and RKO cells were authenticated by the JCRB Cell Bank (National Institute of Biomedical Innovation, Osaka, Japan) using short tandem repeat analysis.

### Establishment of cells exhibiting EMT-dependent RFP expression

To construct an EMT-dependent RFP expression vector, the promoter region and 3′-UTR of the mesenchymal *VIM* gene were cloned from a cDNA template using human normal WI-38 fibroblasts (Supplementary Information Fig. 1). The promoter region and 3′-UTR of the *VIM* gene were amplified by PCR using the following two sets of primers: *VIM* promoter forward, 5′-GGCCATATGGGATCCTTTTTTTCTCCTATCCACTGCAG-3′; *VIM* promoter reverse, 5′-GGGACCGGTTGGCTCCCGGAGAAGAGG-3′; *VIM* 3′-UTR forward, 5′-CGAGCTCACTAGTAAATTGCACACACTCAG-3′; *VIM* 3′-UTR reverse, 5′-GCTCTAGAGAGTTTTTCCAAAGATTTATTGAAG-3′. HCT116 cells were transfected with two types of *VIM* promoter–driven RFP expression vectors without or with the *VIM* 3′-UTR, VR, or VRV3 vector, respectively, using Lipofectamine 3000 reagent (Invitrogen, Carlsbad, CA, USA) and selected using G418 (600 ng/ml). HCT116-VR and HCT116-VRV3 cells were established from 5 candidate clones by evaluating RFP expression after treatment with or without TNF-α (20 ng/ml) for 72 h using a fluorescence microscope (IX71; Olympus, Tokyo, Japan) (Supplementary Information Fig. 2). To determine localization of tumor cells within normal tissues, HCT116-VRV3 cells were transfected with the GFP expression vector; HCT116-VRV3-GFP cells were selected using hygromycin B (400 ng/ml) (Invitrogen). By contrast, RKO cells were transfected with the *VIM* promoter–driven RFP expression vector with *VIM* 3′-UTR (VRV3 vector) and selected using G418 (600 ng/ml). Single-cell clones were established in 96-well plates according to the limiting dilution method. This study was approved by the Recombinant DNA Experiment Safety Committee and carried out in accordance with the approved protocol (Approved ID: 16090).

### Induction of RFP expression in CRC cells in an inflammatory microenvironment

Recombinant human TNF-α, IL-1β, TGF-β, HGF, insulin-like growth factor 1 (IGF-1), EGF, and bFGF were obtained from Sigma-Aldrich (St. Louis, MO, USA). To evaluate EMT-dependent RFP induction after exposure to inflammatory mediators, HCT116 and RKO cells were treated with TNF-α^29^ (20 ng/ml), IL-1β^30^ (1 ng/ml), TGF-β^54^ (10 ng/ml), HGF^55^ (50 ng/ml), IGF-1^56^ (20 ng/ml), EGF^57^ (20 ng/ml), or bFGF^57^ (10 ng/ml) for 48 h. The plasticity of EMT-dependent RFP induction was assessed by replacing with fresh medium after treatment with TNF-α or IL-1β.

### Imaging of CRC cells expressing EMT-dependent fluorescent protein

Time-lapse images of RFP-expressing HCT116 and RKO cells were acquired using an FV10i confocal laser scanning microscope (Olympus, Tokyo, Japan) (Supplementary Movies 1–6).

### Western blot analysis

Whole cell lysates were obtained by using lysis buffer (50 mM Tris-HCl [pH 7.4], 150 mM NaCl, 1% Triton X-100) with a protease inhibitor cocktail (Complete Mini; Roche Applied Science, Mannheim, Germany). After electrophoresis on 6–15% SDS polyacrylamide gels, proteins were transferred onto polyvinylidene difluoride membranes (Hybond-P; GE Health Care, Buckinghamshire, UK). After blocking with Blocking-One reagent (Nacalai Tesque, Kyoto, Japan) at room temperature for 30 min, membranes were incubated with the primary and secondary antibodies. The primary antibodies used were: rabbit anti-RFP (TurboFP635) polyclonal antibody (pAb) (Thermo Fisher Scientific, Fremont, CA, USA), mouse anti–alpha-smooth muscle actin (α-SMA) monoclonal antibody (mAb) (Abcam, Cambridgeshire, UK), rabbit anti-CDH1 mAb, rabbit anti-cytokeratin 20 (CK20) mAb (Cell Signaling Technology, Danvers, MA, USA), and mouse anti–β-actin mAb (Sigma-Aldrich). The secondary antibodies used were: horseradish peroxidase–conjugated antibodies against rabbit IgG or mouse IgG (GE Healthcare). Immunoreactive bands were detected using chemiluminescence substrate (ECL Plus; GE Healthcare).

### ELISA

Levels of extracellular TNF-α and IL-1β in culture medium were analyzed using Quantikine ELISA kits (R&D Systems, Minneapolis, MN, USA).

### Direct co-culture with CRC cells and inflammatory macrophages

To evaluate the EMT-dependent induction of RFP expression in the interplay between CRC and inflammatory cells, HCT116-VRV3 and RKO-VRV3 cells (5 × 10^4^) were co-cultured with RAW264.7 mouse macrophages (5 × 10^4^). Twenty-four h later, lipopolysaccharide (LPS) (200 ng/ml) (Sigma-Aldrich) was added to the culture medium to induce the secretion of TNF-α and IL-1β by RAW264.7 cells. Anti–TNF-α neutralizing antibody (100 ng/ml) and anti–IL-1β neutralizing antibody (500 ng/ml) (Abcam) were added to the culture medium to attenuate the effects of TNF-α and IL-1β secreted by RAW264.7 cells. Then, 48 h later, the images of HCT116-VRV3 and RKO-VRV3 cells co-cultured with RAW264.7 cells were obtained in three randomly selected fields in each group using a confocal laser scanning microscope (FV10i; Olympus). Quantification of RFP expression was analyzed by calculating the integrated densities of RFP with ImageJ software.

### Animal experiments

Animal experimental protocols were approved by the Ethics Review Committee for Animal Experimentation of Okayama University School of Medicine. Six-week-old female BALB/c nude mice (Clea Japan, Tokyo, Japan) were used in this study. The submucosal layer of the rectum was inoculated with HCT116-VRV3-GFP cells (10^6^) suspended in 50 μl of PBS, and the spleen was inoculated with 50 μl of Matrigel (BD Biosciences, San Diego, CA, USA) containing 10^6^ cells, as reported previously^33,34^. Seven days after inoculation, rectum and liver tissues were dissected to examine rectal and metastatic liver tumors. Twenty-one days after inoculation, liver tissues were dissected to examine metastatic liver tumors.

### Immunohistochemistry

Rectal and liver tissues containing tumors were fixed in 4% paraformaldehyde solution. Tissue sections (3 μm) were examined by immunohistochemistry. After deparaffinization, rehydration, and antigen retrieval, tissue sections were incubated overnight with the primary antibodies: rabbit anti-GFP pAb (5 μg/ml) (Invitrogen), FITC-labeled rabbit anti-RFP pAb (2 μg/ml) (Abcam), rabbit anti-CD68 pAb (1 μg/ml) (Abcam), and mouse anti–IL-1β mAb (1:100) (Cell Signaling Technology). The sections were then incubated with secondary antibodies: Alexa 488–labeled goat antibody against mouse IgG and Alexa-568–labeled goat antibody against rabbit IgG for 1 h. The nuclei were stained with 4′,6-diamidino-2-phenylindole (DAPI; 100 μg/ml) solution. Immunofluorescence-positive cells were imaged using a LSM780 confocal laser scanning microscope (Carl Zeiss, Jena, Germany).

### Statistical analysis

Data are shown as mean ± SD. Significance of differences in two groups was assessed using the Student’s *t*-test. Significance of differences in multiple groups was assessed using a one-way ANOVA followed by Tukey HSD multiple comparison test. Statistical analysis was performed with SPSS statistics software (version 22). *P* < 0.05 was considered indicative of significance.

## Supplementary information

Supplementary Movie 1

Supplementary Movie 2

Supplementary Movie 3

Supplementary Movie 4

Supplementary Movie 5

Supplementary Movie 6

Supplementary Information
