# Supplementary material for: Visualization of epithelial-mesenchymal transition in an inflammatory microenvironment–colorectal cancer network
Source: Sci Rep. 2019 Nov 8;9:16378. doi: 10.1038/s41598-019-52816-z (PMC6841984; doi:10.1038/s41598-019-52816-z)
Supplement: Supplementary file 7 — Supplementary Information [file 41598_2019_52816_MOESM7_ESM.pdf]

Supplementary Information

Visualization of epithelial-mesenchymal transition in inflammatory microenvironment-colorectal cancer network

Takeshi Ieda[1], Hiroshi Tazawa[1,3], Hiroki Okabayashi[1], Shuya Yano[1], Kunitoshi Shigeyasu[1], Shinji Kuroda[1,3], Toshiaki Ohara[1,2], Kazuhiro Noma[1], Hiroyuki Kishimoto[1,4], Masahiko Nishizaki[1], Shunsuke Kagawa[1,4], Yasuhiro Shirakawa[1], Takashi Saitou[5], Takeshi Imamura[5] and Toshiyoshi Fujiwara[1]

Department of [1]Gastroenterological Surgery, [2]Pathology & Experimental Medicine, Okayama University Graduate School of Medicine, Dentistry and Pharmaceutical Sciences, Okayama 700-8558, Japan. [3]Center for Innovative Clinical Medicine and [4]Minimally Invasive Therapy Center, Okayama University Hospital, Okayama 700-8558, Japan. [5]Department of Molecular Medicine for Pathogenesis, Ehime University Graduate School of Medicine, Ehime 791-0295, Japan.

Supplementary Materials

Supplementary Information Figure 1
Scheme for construction of VR and VRV3 vector plasmids

Supplementary Information Figure 2
Evaluation of RFP expression in HCT116-VR and HCT116-VRV3 clones treated with or without TNF-α.

Supplementary Information Figure 3
Comparison of morphology and cell proliferation in parental and VRV3 vector-transfected CRC cells

Supplementary Information Figure 4
Induction of RFP expression in association with EMT phenotype in HCT116-VRV3 cells treated with TNF-α or IL-1β

Supplementary Information Figure 5
Induction of RFP expression in association with EMT phenotype in RKO-VRV3 cells treated with TNF-α or IL-1β

Supplementary Information Figure 6
Suppression of TNF-α-induced RFP expression in CRC cells by treatment with anti-TNF-α neutralizing antibody

Supplementary Information Figure 7
Suppression of IL-1β-induced RFP expression in CRC cells by treatment with anti-IL-1β neutralizing antibody

Supplementary Information Figure 8
Time-lapse imaging of RFP expression in CRC cells in indirect co-culture with LPS-stimulated RAW264.7 cells

Supplementary Information Figure 9
M1/M2 polarization of RAW264.7 cells by treatment with LPS and IL-4

Supplementary Materials

Supplementary Information Movie 1
Time-lapse imaging of non-treated HCT116-VRV3 cells for 48 h.

Supplementary Information Movie 2
Time-lapse imaging of HCT116-VRV3 cells treated with TNF-α for 48 h.

Supplementary Information Movie 3
Time-lapse imaging of HCT116-VRV3 cells treated with IL-1β for 48 h.

Supplementary Information Movie 4
Time-lapse-imaging of non-treated RKO-VRV3 cells for 48 h.

Supplementary Information Movie 5
Time-lapse imaging of RKO-VRV3 cells treated with TNF-α for 48 h.

Supplementary Information Movie 6
Time-lapse imaging of RKO-VRV3 cells treated with IL-1β for 48 h.

Supplementary Methods

Cell proliferation assay. Parental and VRV3 vector-transfected HCT116 or RKO cells were seeded at a density of 10^4 cells in 24-well tissue culture plates. Twenty-four h later, cells were counted every day for 3 days. The average number of cells was determined at each time point in triplicate. The morphology of each cell was observed under an inverted microscope (IX71; Olympus, Tokyo, Japan).

Flow cytometric analysis. HCT116-VRV3 and RKO-VRV3 cells, seeded 3 x 10^5 cells/dish in 100-mm dishes, were incubated without or with TNF-α (20 ng/ml) or IL-1β (1 ng/ml) for 48 h. The cells were trypsinized and were analyzed using a flow cytometry (FACS Array; Becton Dickinson, Sab Jose, CA, USA).

Quantification of RFP-expressing CRC cells by microplate reader. HCT116-VRV3 and RKO-VRV3 cells, seeded 3 x 10^5 cells/dish in 100-mm dishes, were incubated without or with TNF-α (20 ng/ml) or IL-1β (1 ng/ml) for 48 h. The cells were trypsinized, suspended in PBS containing 2% FBS, and seeded in 96-well black plate at a density of 4 x 10^5 cells/well. The fluorescence intensity of RFP-positive cells was analyzed by the SpectraMax i3 multi-mode microplate reader (Molecular Devices, Sunnyvale, USA). The relative quantification of RFP-expressing CRC cells was calculated in reference to the RFP intensity of non-treated CRC cells.

Quantitative real-time reverse transcription-PCR analysis. Total RNA was extracted from cells by using a miRNeasy Mini Kit (Qiagen, Valencia, CA, USA). After synthesis of cDNA using 100 ng of total RNA, the mRNA expression of *CDH1* and *VIM* was determined by quantitative reverse transcription-polymerase chain reaction using the StepOnePlus™ real-time PCR system (Applied Biosystems, Foster City, CA, USA). The relative expression levels were calculated using the 2^{-DDCt} method after normalization with reference to *glyceraldehydes-3-phosphate dehydrogenase* mRNA expression.

Migration and invasion assay. The 24-well Transwell chamber plate with an 8 μm pore size filter membrane (BD Bioscience, Bedford, MA, USA) was used. HCT116-VRV3 (5 x 10^4 cells) or RKO-VRV3 cells (2 x 10^5 cells) were seeded in serum-free medium in the top chamber with the non-coated or Matrigel-coated membrane for migration or invasion assay, respectively. The medium supplemented with 10% FBS as a chemoattractant was added in the lower chamber. After incubation with 24 h (HCT116-VRV3 cells) and 48 h (RKO-VRV3 cells), the cells on the lower surface of the membrane were fixed and stained with crystal violet. The number of the cells migrating through the membrane was counted under a light microscope.

Indirect co-culture with CRC cells and inflammatory macrophages. To evaluate the involvement of cytokines secreted from inflammatory cells in the EMT induction of CRC cells, we used a 2-well culture insert (ibidi, Martinsried, Germany), in which HCT116-VRV3 or RKO-VRV3 cells and RAW264.7 cells pre-stained with CellTracker Green (Thermo Fisher Scientific, Fremont, CA, USA) were seeded at a density of 10^4 cells/well. Lipopolysaccharide (LPS) (200 ng/ml) (Sigma-Aldrich) was administered in the culture medium to induce the secretion of TNF-α and IL-1β from RAW264.7 cells. Time-lapse images of RFP-expressing HCT116-VRV3 cells were acquired using a FV10i confocal laser scanning microscope (Olympus).

Induction of M1 and M2 macrophages from RAW264.7 cells. To obtain the M1 and M2 macrophages, mouse macrophage RAW264.7 cells were treated with LPS (200 ng/ml) and IL-4 (20 ng/ml) for 48h. The morphology of RAW264.7 cells non-treated or treated with LPS or IL-4 was observed under an inverted microscope (IX71; Olympus). Whole cell lysate for non-treated, LPS-treated, and IL-4-treated RAW264.7 cells were prepared and subjected to western blot analysis to analyze the expression of macrophage marker, CD68 and CD204 proteins, by using rabbit anti-CD68 mAb (Abcam, Cambridgeshire, UK) and mouse anti-CD204 mAb (Trans Genic Inc., Fukuoka, Japan), respectively. Moreover, IL-4-treated RAW264.7 cells were stained with CellTracker Green and used in direct co-culture with HCT116-VRV3 cells.

Direct co-culture with CRC and M2 macrophage. HCT116-VRV3 cells (5 x 10^4 cells) were co-cultured with M2 macrophages derived from mouse macrophage RAW264.7 cells (5 x 10^4 cells). IL-4 (20 ng/ml) (Sigma-Aldrich) was administered in the culture medium to induce the M2-polarized macrophages from RAW264.7 cells.

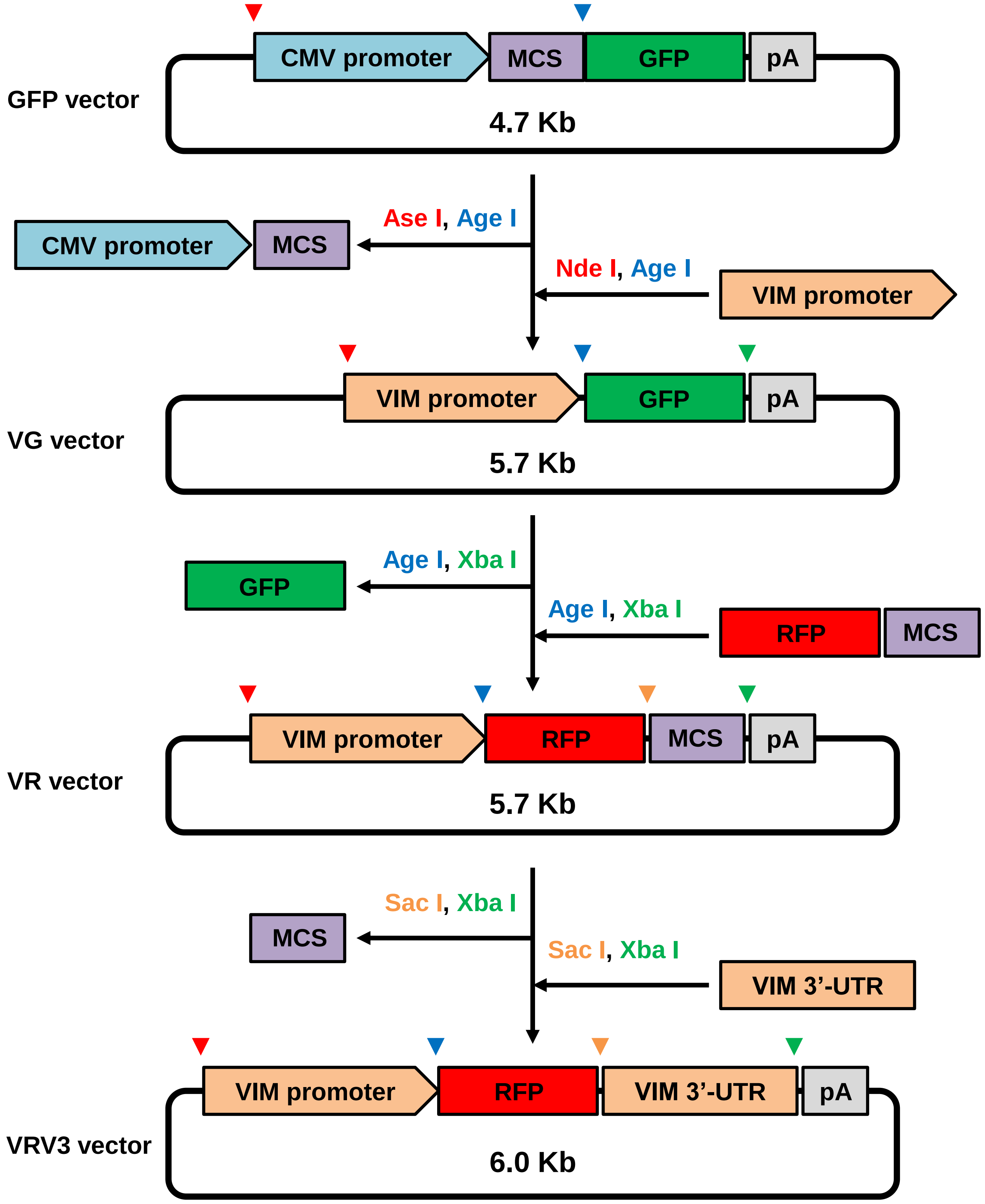

Supplementary Information Figure 1

Scheme for construction of VR and VRV3 vector plasmids. The VR and VRV3 vectors were constructed from GFP vector by inserting the RFP cording region and the promoter region and 3' UTR of VIM gene.

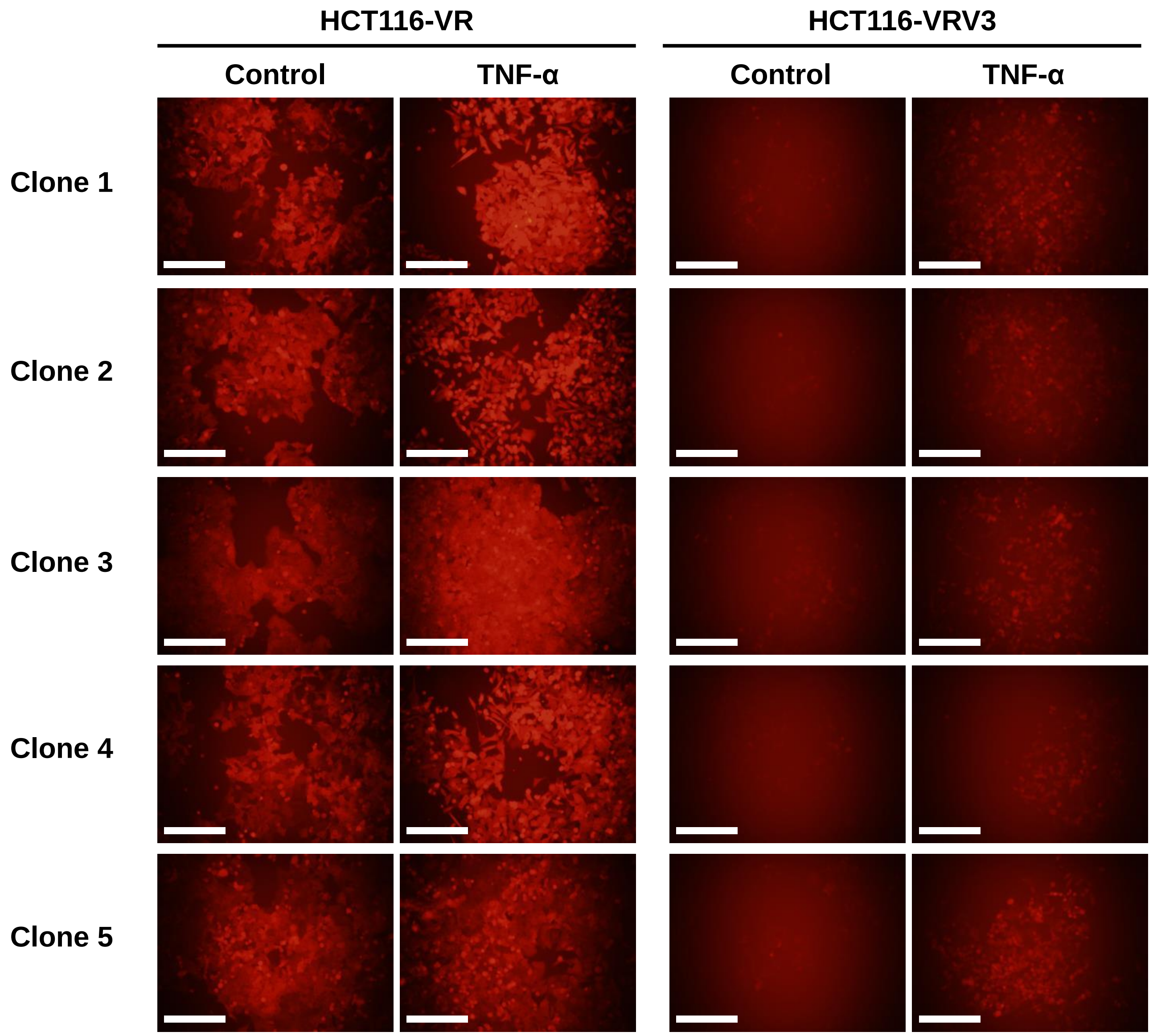

Supplementary Information Figure 2

Evaluation of RFP expression in HCT116-VR and HCT116-VRV3 clones after treatment with or without TNF-α (20 ng/ml) for 72 h. RFP expression were analyzed using a fluorescence microscope (IX71; Olympus). Scale bars: 200 μm.

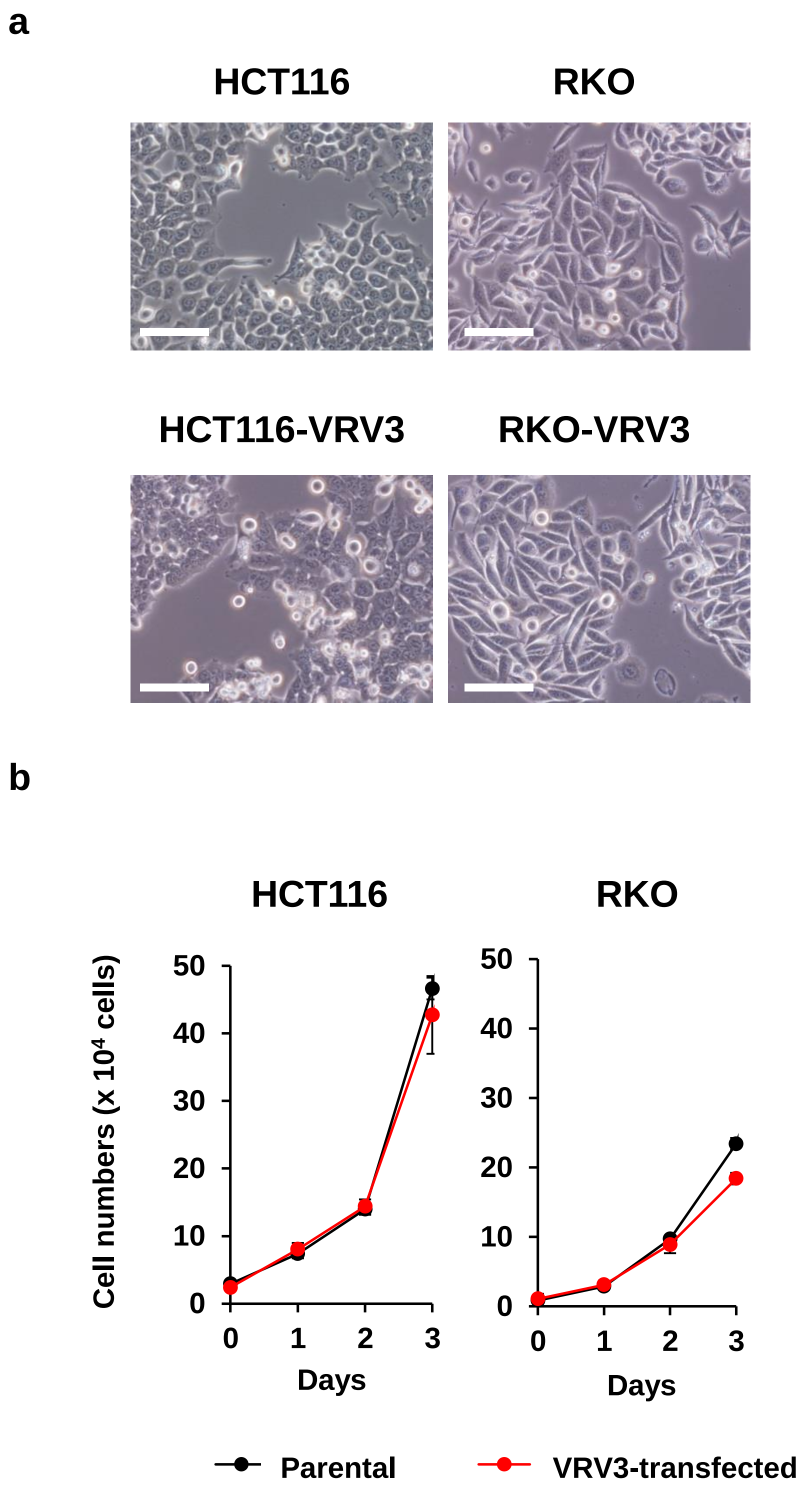

Supplementary Information Figure 3

Comparison of morphology and cell proliferation in parental and VRV3 vector-transfected CRC cells. Parental and VRV3 vector-transfected HCT116 and RKO cells were seeded in 24-well culture plates at a density of 10^4 cells/well. (a) The morphology of each cell was observed under an inverted microscope (IX71; Olympus). Scale bars: 100 µm. (b) The proliferation of each cell was analyzed by counting the number of each cell every day for 3 days. Data are expressed as mean values ± SD (n = 3).

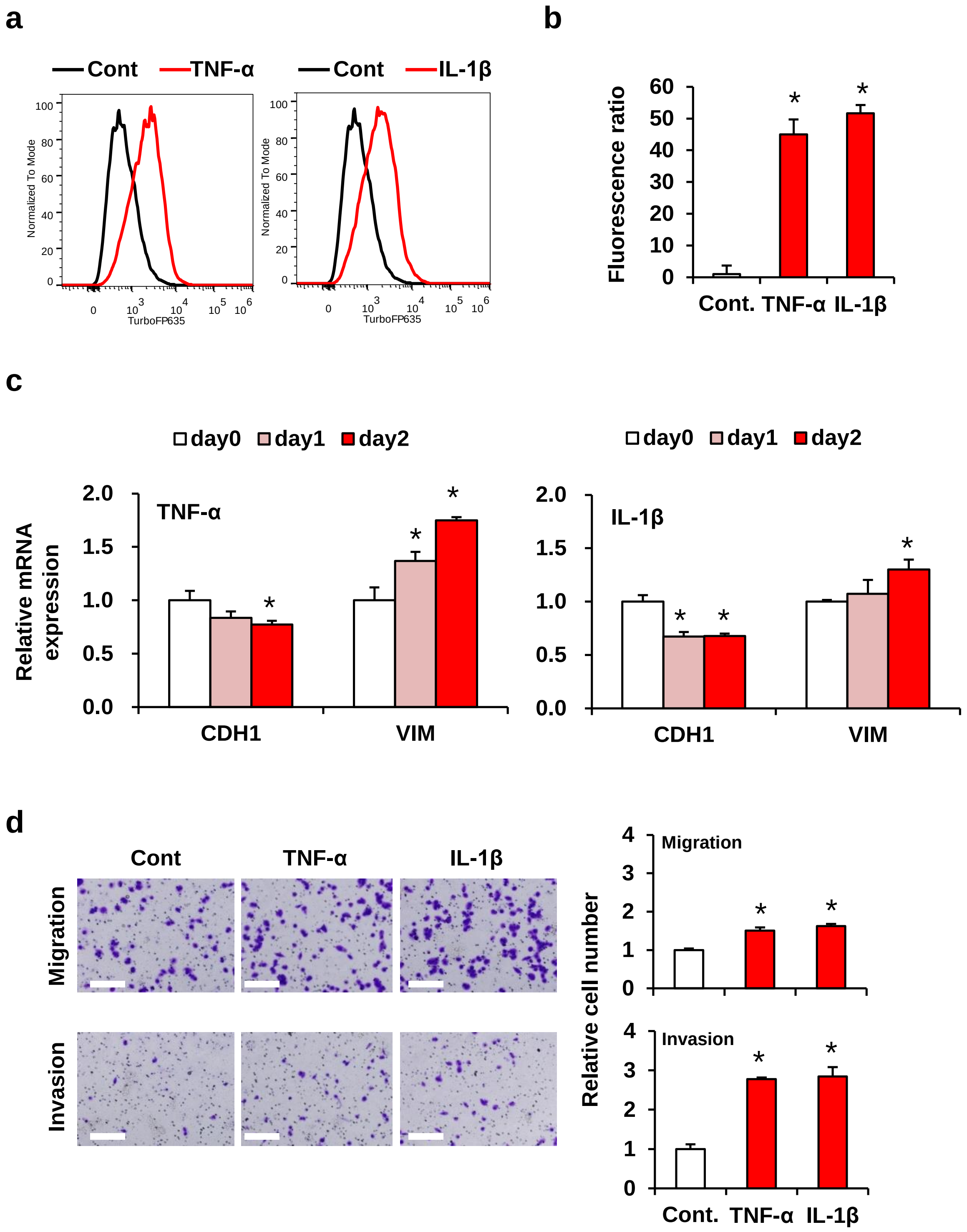

Supplementary Information Figure 4

Induction of RFP expression in association with EMT phenotype in HCT116-VRV3 cells treated with TNF-α or IL-1β. HCT116-VRV3 cells were treated with TNF-α (20 ng/ml) or IL-1β (1 ng/ml) for 48h. (a, b) The red fluorescence intensity was analyzed using flow cytometry (a) and microplate reader (b). (c) The mRNA expression of epithelial marker *CDH1* gene and mesenchymal marker *VIM* gene was analyzed by qRT-PCR. (d) The migration and invasion abilities were analyzed using Transwell chamber plates. Scale bars: 100 μm. Data are expressed as mean values ± SD (n = 3). *: $P < 0.05$ (vs cont or day0).

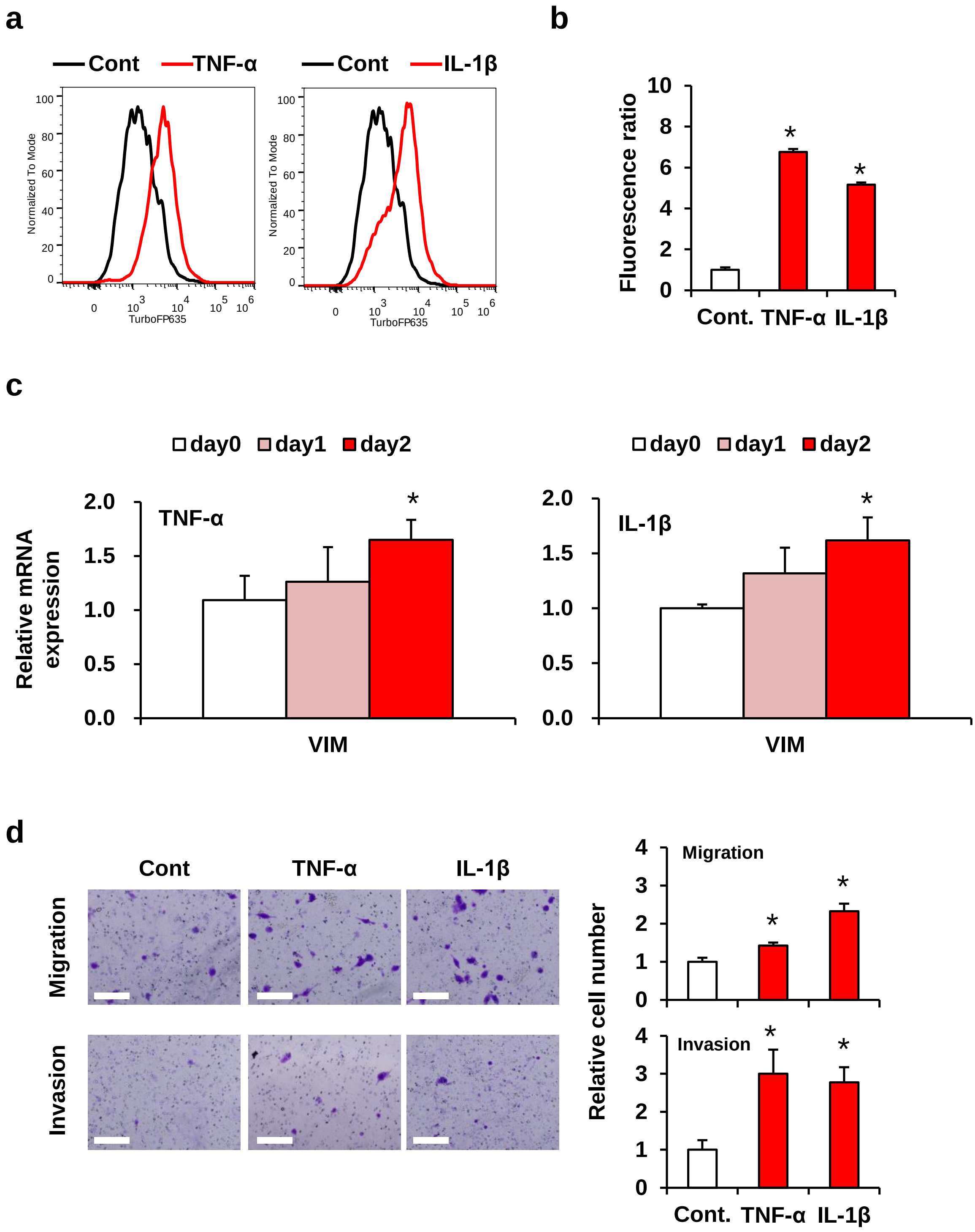

Supplementary Information Figure 5

Induction of RFP expression in association with EMT phenotype in RKO-VRV3 cells treated with TNF-α or IL-1β. RKO-VRV3 cells were treated with TNF-α (20 ng/ml) or IL-1β (1 ng/ml) for 48h. (a, b) The red fluorescence intensity was analyzed using flow cytometry (a) and microplate reader (b). (c) The mRNA expression of mesenchymal marker *VIM* gene was analyzed by qRT-PCR. (d) The migration and invasion abilities were analyzed using Transwell chamber plates. Scale bars: 100 µm. Data are expressed as mean values ± SD (n = 3). *: $P < 0.05$ (vs cont or day0).

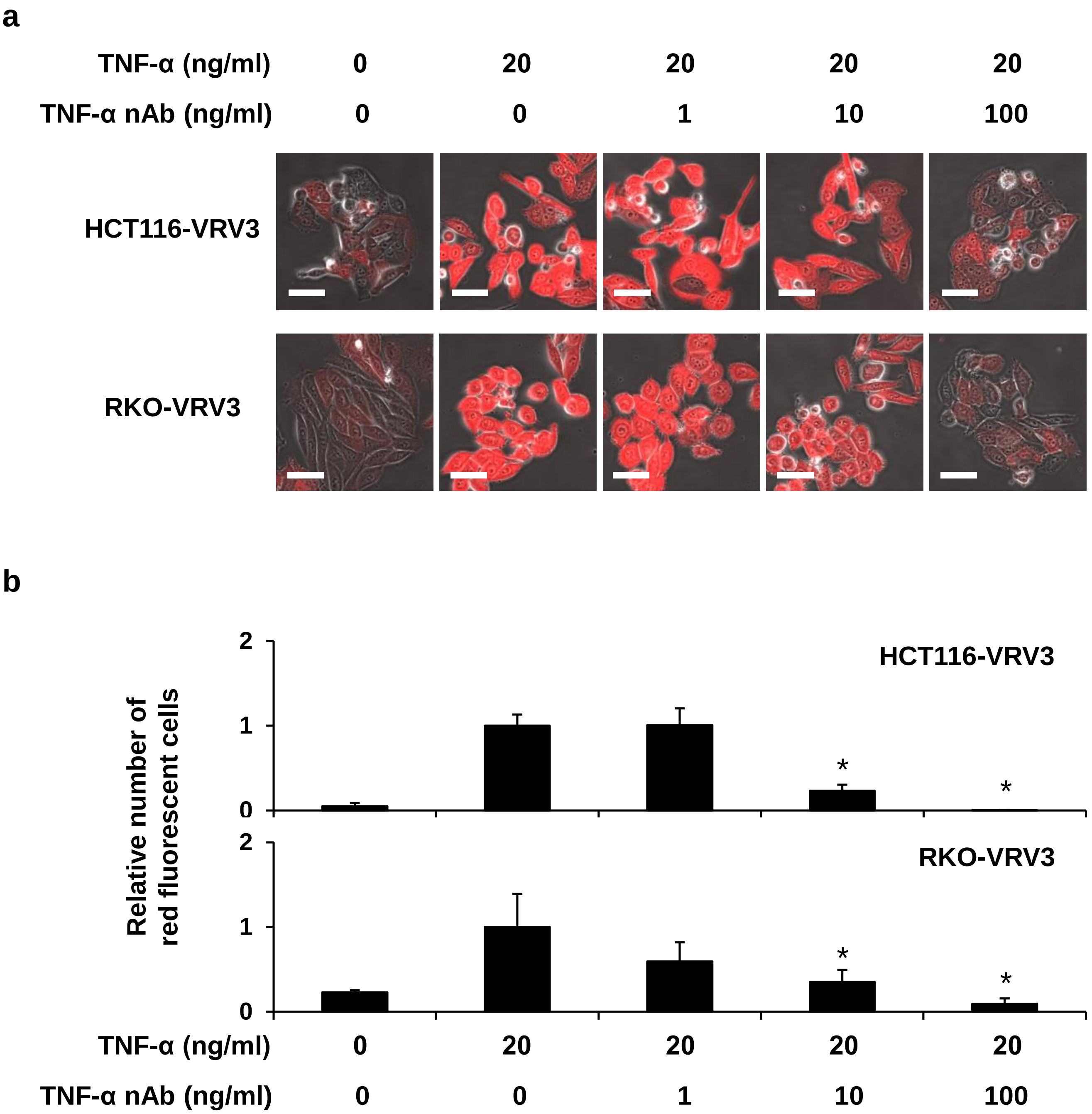

Supplementary Information Figure 6

Suppression of TNF-α-induced RFP expression in CRC cells by treatment with anti-TNF-α neutralizing antibody. HCT116-VRV3 and RKO-VRV3 cells were treated without or with TNF-α (20 ng/ml) for 48 h in the presence of anti-TNF-α neutralizing antibody at various doses (1, 10, 100 ng/ml). (a) The morphology and RFP expression were analyzed using a FV10i confocal laser scanning microscope (Olympus). Scale bars: 50 μm. (b) The fluorescence intensity of RFP-positive cells was analyzed by the SpectraMax i3 multi-mode microplate reader (Molecular Devices). Data are expressed as mean values ± SD (n = 3). *: $P < 0.05$ (vs TNF-α treatment).

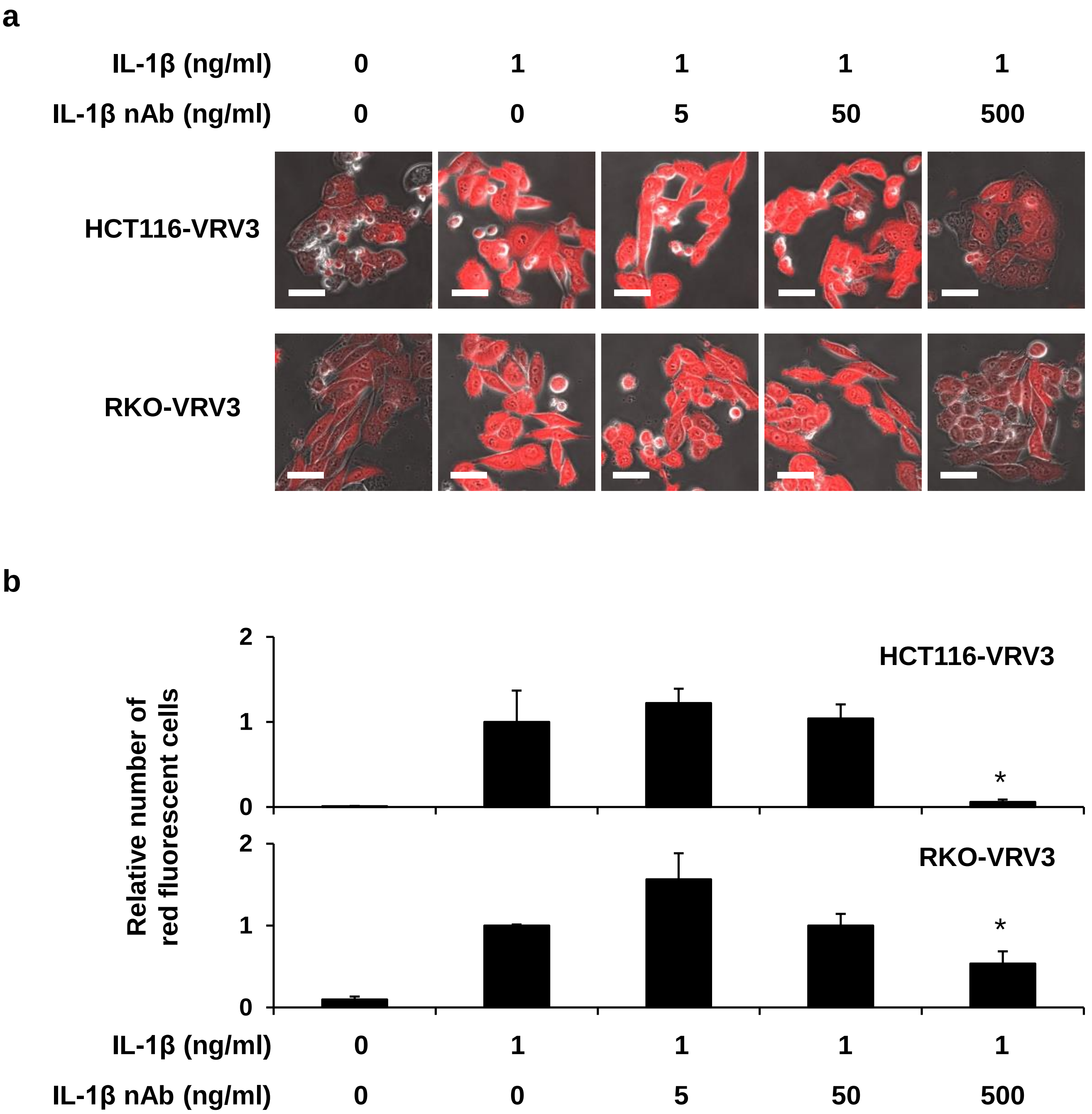

Supplementary Information Figure 7

Suppression of IL-1β-induced RFP expression in CRC cells by treatment with anti-IL-1β neutralizing antibody. HCT116-VRV3 and RKO-VRV3 cells were treated without or with IL-1β (20 ng/ml) for 48 h in the presence of anti-IL-1β neutralizing antibody at various doses (5, 50, 500 ng/ml). (a) The morphology and RFP expression were analyzed using a FV10i confocal laser scanning microscope (Olympus). Scale bars: 50 μm. (b) The fluorescence intensity of RFP-positive cells was analyzed by the SpectraMax i3 multi-mode microplate reader (Molecular Devices). Data are expressed as mean values ± SD (n = 3). *: $P < 0.05$ (vs IL-1β treatment).

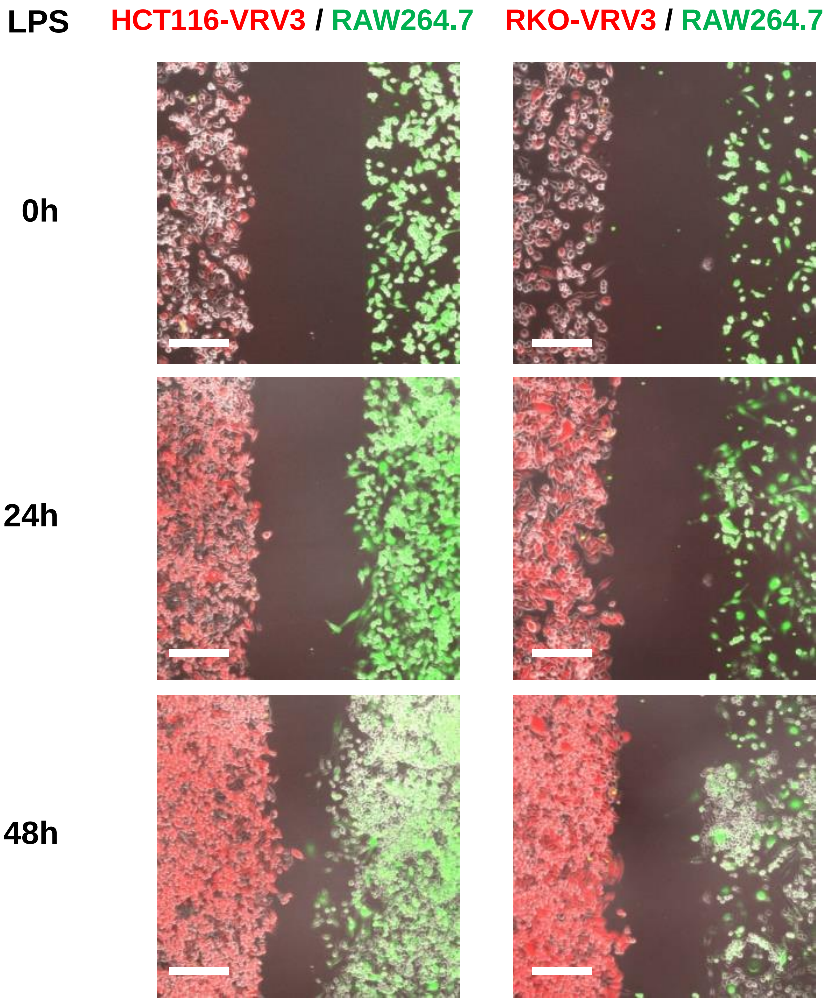

Supplementary Information Figure 8

Time-lapse imaging of RFP expression in CRC cells in indirect co-culture with LPS-stimulated RAW264.7 cells. HCT116-VRV3 or RKO-VRV3 cells and RAW264.7 cells pre-stained with CellTracker Green were seeded at a density of 10^4 cells/well in a 2-well culture insert (ibidi). Twenty-four later, LPS (200 ng/ml) was administered in the culture medium. Time-lapse images of RFP-expressing HCT-VRV3 or RKO-VRV3 cells with green-colored RAW264.7 cells were acquired using a FV10i confocal laser scanning microplate (Olympus). Scale bars: 50 μm.

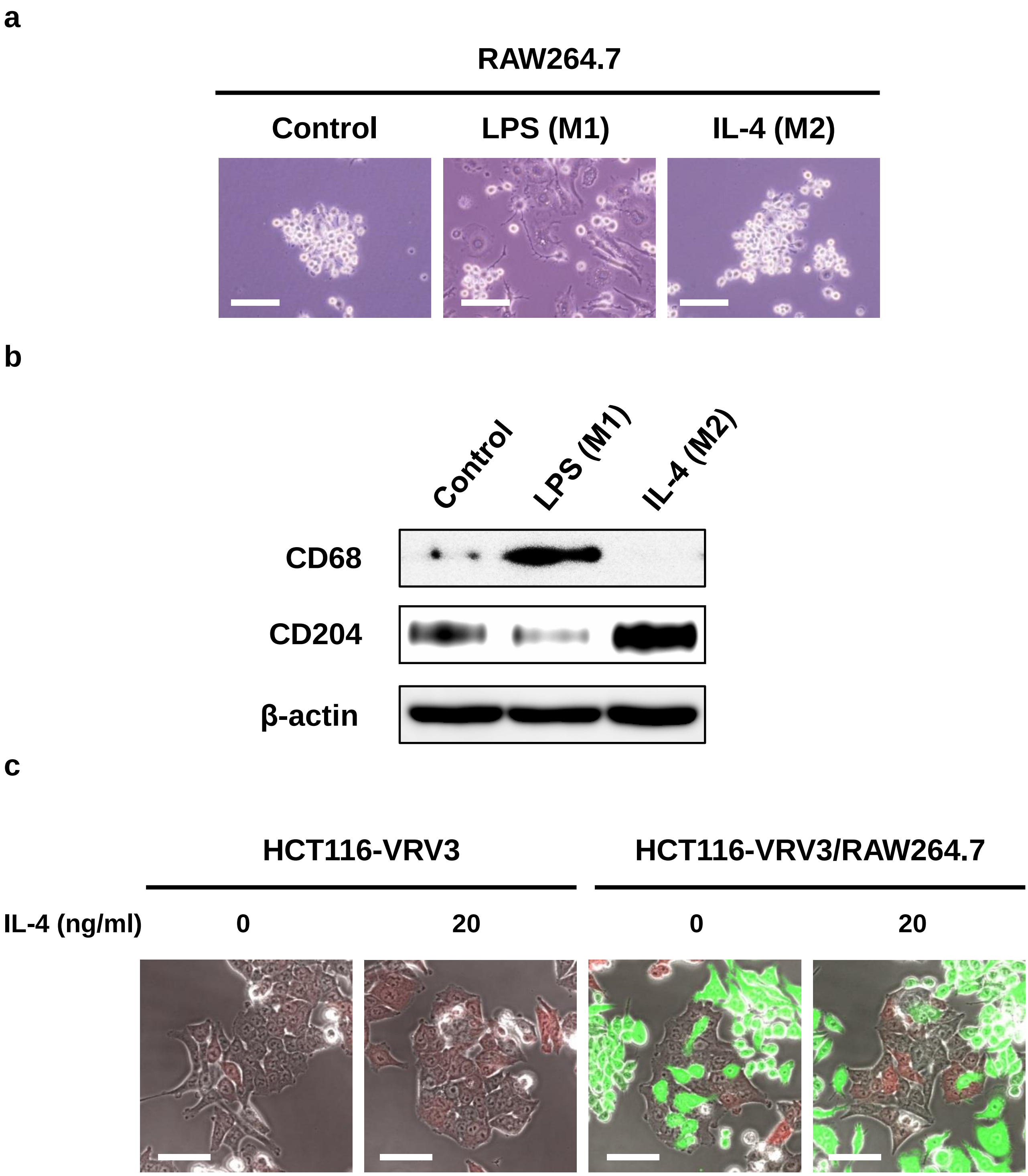

Supplementary Information Figure 9

M1/M2 polarization of RAW264.7 cells by treatment with LPS and IL-4. RAW264.7 cells were treated with LPS (200 ng/ml) or IL-4 (20 ng/ml) for 48 h. (a) The morphology of RAW264.7 cells untreated (control) or treated with LPS (M1) or IL-4 (M2). Scale bars: 100 μm. (b) The protein expression of macrophage marker CD68 and M2 macrophage marker CD204 in RAW264.7 cells untreated (control) or treated with LPS (M1) or IL-4 (M2). (c) Direct co-culture of HCT116-VRV3 cells without or with RAW264.7 cells in the presence of IL-4 (10 ng/ml). Scale bars: 50 μm.
